# The informative value and design of orthodontic practice websites in The Netherlands

**DOI:** 10.1186/s40510-019-0302-0

**Published:** 2020-01-13

**Authors:** Cesar Guy Oey, Christos Livas

**Affiliations:** Department of Orthodontics, Academic Centre for Dentistry Amsterdam (ACTA), University of Amsterdam and VU University Amsterdam, Gustav Mahlerlaan 3004, 1081 LA Amsterdam, The Netherlands

**Keywords:** Websites, Practice management, Ethics, Patient education

## Abstract

**Background:**

The aims of this cross-sectional study were to investigate the regulatory compliance of Dutch practice websites offering orthodontic services, readability of the available treatment information, website design as well as possible relationship with practice location and professional qualification of practitioners.

**Methods:**

A comprehensive Internet search was performed using the Google search engine and five relevant terms in Dutch. Eligibility screening of the first 50 results of each search led to the final inclusion of 111 websites. The content of the selected websites was evaluated in terms of compliance to international regulations on ethical advertising guidelines (CED), treatment information text readability using Flesch Reading Ease Score (FRES), and website design using the BDC assessment tool.

**Results:**

Reporting of websites according to CED guidelines covered on average 85% of the mandatory items. No significant differences were observed between dental and orthodontic practices, and between practices located in densely and sparsely populated regions (*P* > 0.05). The mean FRES of the displayed information indicated difficult-to-understand text. BDC scores of multi-location practices were significantly higher than the rest (*P* < 0.006).

**Conclusions:**

The websites of orthodontic practices in The Netherlands do not fully comply with CED guidelines on ethical advertising. Readability of the displayed information and website technical performance needs to be further optimized.

## Background

The practice website is nowadays considered an effective marketing and communication tool for a thriving orthodontic practice. Orthodontic practices with websites and active engagement through social media were positively related to new patient starts per year [1]. Online presence of the practice enables the spread of information to current and future patients regarding practice location, facilities, staff, and provided services while contacting the practice team is frequently initiated through the website [2].

As the content of health-related websites has been criticized for extreme variability in quality, imbalance between posted information and patient health literacy, and likely interference of commercial interests, regulatory action has been taken at European Union (EU) and national level to optimize the development of dental practice websites [3]. The Council of European Dentists has published the Code of Ethics for Dentists (CED), a code including mandatory and discretionary provider and professional information that should be displayed on a dental website to guide commercial communications of dentists within EU and information services on the Internet [4]. In line with European guidance, the General Dental Council (GDC) in the UK has produced an ethical framework for regulating dental advertising by practice websites [5]. However, compliance of UK dental and orthodontic practices to GDC core principles is generally poor and rather slowly improving [2, 6–9].

To this end, the Royal Dutch Dental Association (KNMT: Koninklijke Nederlandse Maatschappij tot bevordering der Tandheelkunde), the professional association for dentists, oral surgeons, and orthodontists in The Netherlands, has recommended a 7-point checklist regarding the desired information available on practice websites [10]. Using a 60-item questionnaire developed by the authors, the content of Dutch dental practice websites was found to be highly variable. Strikingly, essential details like dentist’s name, professional title, and registration number, e-mail address and last update date were often missing from the websites of Dutch dental practices [11].

Due to the widespread acceptance of orthodontics by the general Dutch population and shortage of practicing orthodontists, especially in non-metropolitan areas, 27–40% of the orthodontic caseload is being served by dentists [12, 13]. Given practice-specific parameters like location (i.e., urban vs. rural areas), ownership status (i.e., multi-owner vs. single-owner practice), and professional title (i.e., dentists vs. orthodontists) may have a certain impact on the website’s quality [14], it would be interesting to investigate the informational content of the websites of the practices of orthodontic treatment providers in The Netherlands. Thus, the aims of this study were to examine the compliance of Dutch orthodontic practices to EU regulations (CED), readability of the posted orthodontic information, website design, and possible relationship of the aforementioned website features with practice location and professional qualification of practitioners.

## Methods

### Search strategy

Using Google (www.google.com), an Internet search was carried out on April 17, 2019. This decision was made on the basis of the high search validity and popularity of the Google search engine in The Netherlands [15, 16]. Prior to initiating search, browser history, cookies, and cache were cleared. Five terms in Dutch were originally identified to yield the most Google results and subsequently used for the purposes of the study: “orthodontics,” “orthodontic practice,” “orthodontist’s practice,” “practice of orthodontists,” and “dentist for orthodontics.” Dutch professional associations have recently agreed upon using the title “dentist for orthodontics” by dentists practicing orthodontics to avoid confusion for patients. To outperform the standard online search behavior of a layperson limited to the first 8–10 results, the first 5 pages, were considered for eligibility [17–19]. Only websites of orthodontic practices, and dental practices providing orthodontic treatment located in the Netherlands were included.

### Website assessment

#### Regulatory compliance

Practice information was critically reviewed for compliance to CED by one observer (first author). The review focused on reporting mandatory information such as name and geographic address, contact details of the service provider, professional title and the country the title was obtained, license, and registration information. Websites were screened for optional CED items, namely opening and contact hours of the practice, details of emergency care, information about treatment techniques as well as links to professional associations like the Dutch Association of Orthodontists (NVvO; Nederlandse Vereniging van Orthodontisten) and the Orthodontic Association of General Practitioners (OVAP; Orthodontische Vereniging van Algemeen Practici).

#### Readability of orthodontic information

Text readability was evaluated by means of the Flesch Reading Ease Score (FRES) (Flesch 1948). FRES is an objective measurement of the reading level required by the reader to fully understand the text. The formula to calculate FRES is as follows: 206.835—(1.015 × Average number of words per sentence)—(84.6 × Average number of syllables per word). FRES ranges from 0 to 100, where a higher score indicates an easier-to-read text. Scores are categorized as follows: 0–29 very confusing, 30–49 difficult, 50–59 fairly difficult, 60–69 standard, 70–79 fairly easy, 80–89 easy, and 90–100 very easy. A sample text of 200 to 500 words describing orthodontic appliances was extracted and tested using the Automatic Readability Checker, a free online text readability consensus calculator (http://www.readabilityformulas.com/free-readability-formula-tests.php).

#### Design

To evaluate the form and function of websites, a free online website assessment tool (https://www.bdc.ca/en/articles-tools/entrepreneur-toolkit/business-assessments/pages/free-website-evaluation.aspx), developed by bdc* (bdc*, Montreal, Quebec, Canada), was utilized. After entering the website URL, an overall score lying between 0 and 100 was generated, based on aspects including social media integration, web-optimized images, mobile speed, mobile optimization, search terms, and amount of content.

The collected data on CED reporting, FRES, and BDC scores were classified by provider’s professional title (i.e., orthodontist, dentist or combination in case of multiple practitioners) and practice location (i.e., in the most three most densely populated provinces; North Holland, South Holland, and Utrecht, the rest of the provinces, and in more than one provinces).

### Statistical analysis

The presence of CED information was recorded in percentages, and differences between groups defined by practice location and professional qualification were assessed using chi-squared tests. For the numerical data, that is FRES and BDC scores, means, standard deviations, and ranges were calculated and differences between groups were tested using one-way analysis of variance (ANOVA). Statistical significance was set at 0.05. All statistical analyses were conducted using IBM SPSS Statistics software version 25 (IBM Corp., Armonk, NY, USA).

## Results

**Fig. 1:**
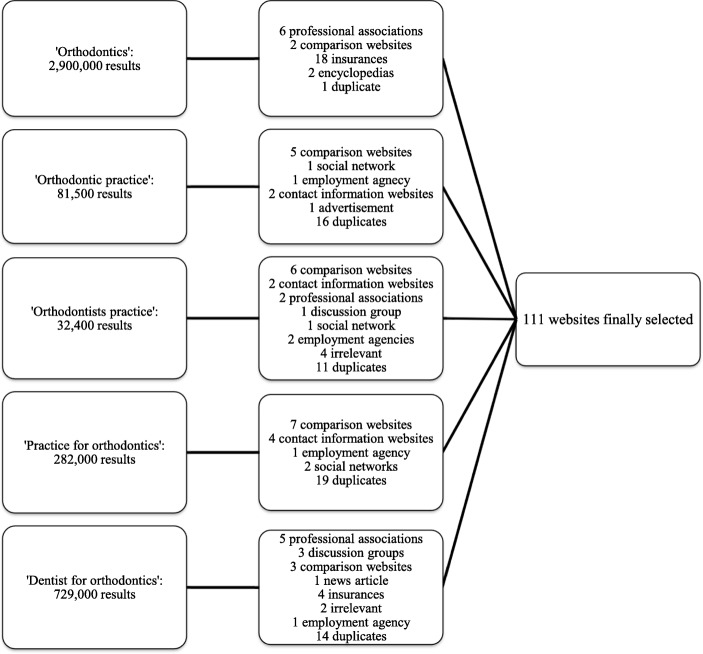
Flowchart diagram of the selection process

The search term combination yielded 4,024,900 results that were initially refined to 250 results. After applying the exclusion criteria for website type, a total of 111 websites were considered eligible (Fig. 1). Sixty-eight (62%) of the websites belonged to orthodontic practices, and 28 (25%) to dental practices offering orthodontic services.

### Reporting of mandatory CED information

On average, the websites complied with 6.8 out of the 8 mandatory CED items. All websites regardless of classification revealed practice name, address, and phone number. The lowest reporting rates were found overall for the provider’s professional registration (41%) and country of education in multi-location and multi-disciplinary practices (20 and 27%, respectively) (Table 1). Websites of all practices established in multiple provinces reported 5 out of 8 mandatory CED items. No statistically significant differences were observed in reporting either between websites of orthodontic, dental and multi-disciplinary practices or websites of practices as classified by location (*P* > 0.05).

**Table 1:** Reporting of mandatory information items by the examined websites according to CED guidelines (in parentheses, the respective percentages)

		Professional title			Practice location		
	Overall	Orthodontist	Dentist	Combination	NH, SH, U	Other provinces	Multiple provinces
Provider’s name	103 (93%)	65 (96%)	27 (96%)	10 (91%)	51 (98%)	47 (87%)	5 (100%)
Practice address	111 (100%)	68 (100%)	28 (100%)	11 (100%)	52 (100%)	54 (100%)	5 (100%)
E-mail	102 (92%)	64 (94%)	26 (93%)	10 (91%)	50 (96%)	47 (87%)	5 (100%)
Phone number	111 (100%)	68 (100%)	28 (100%)	11 (100%)	52 (100%)	54 (100%)	5 (100%)
Origin of education	65 (59%)	54 (79%)	8 (29%)	3 (27%)	32 (62%)	32 (60%)	1 (20%)
Registration number	45 (41%)	32 (47%)	9 (32%)	4 (36%)	21 (52%)	20 (37%)	4 (80%)
Practice name	111 (100%)	68 (100%)	28 (100%)	11 (100%)	52 (100%)	54 (100%)	5 (100%)

NH, SH, and U stand for the provinces of North Holland, South Holland, and Utrecht

#### Reporting of discretionary CED information

Practice opening hours were listed by 102 out of 111 websites (92%), followed by information regarding appliances and techniques (80%), emergency contact details (64%), and professional association links (57%) (Table 2). Practices located in North Holland, South Holland, and Utrecht were significantly more likely to list emergency care information (*P* = 0.02), and orthodontists provided significantly more links to a professional association than the other providers (*P* = 0.0004).

**Table 2:** Reporting of discretionary information items by the examined websites according to CED guidelines (in parentheses, the respective percentages)

		Professional title			Practice location		
	Overall	Orthodontist	Dentist	Combination	NH, SH, U	Other provinces	Multiple provinces
Opening hours	102 (92%)	61 (90%)	27 (96%)	11 (100%)	49 (94%)	48 (89%)	5 (100%)
Emergency care	71 (64%)	42 (62%)	22 (79%)	11 (100%)	40 (77%)	29 (54%)	5 (100%)
Treatment information	89 (80%)	57 (84%)	21 (75%)	9 (82%)	45 (87%)	40 (74%)	4 (80%)
Link to professional associations	63 (57%)	51 (75%)	8 (29%)	4 (36%)	28 (54%)	34 (63%)	1 (20%)

NH, SH, and U stand for the provinces of North Holland, South Holland, and Utrecht

#### Readability

**Fig. 2:**
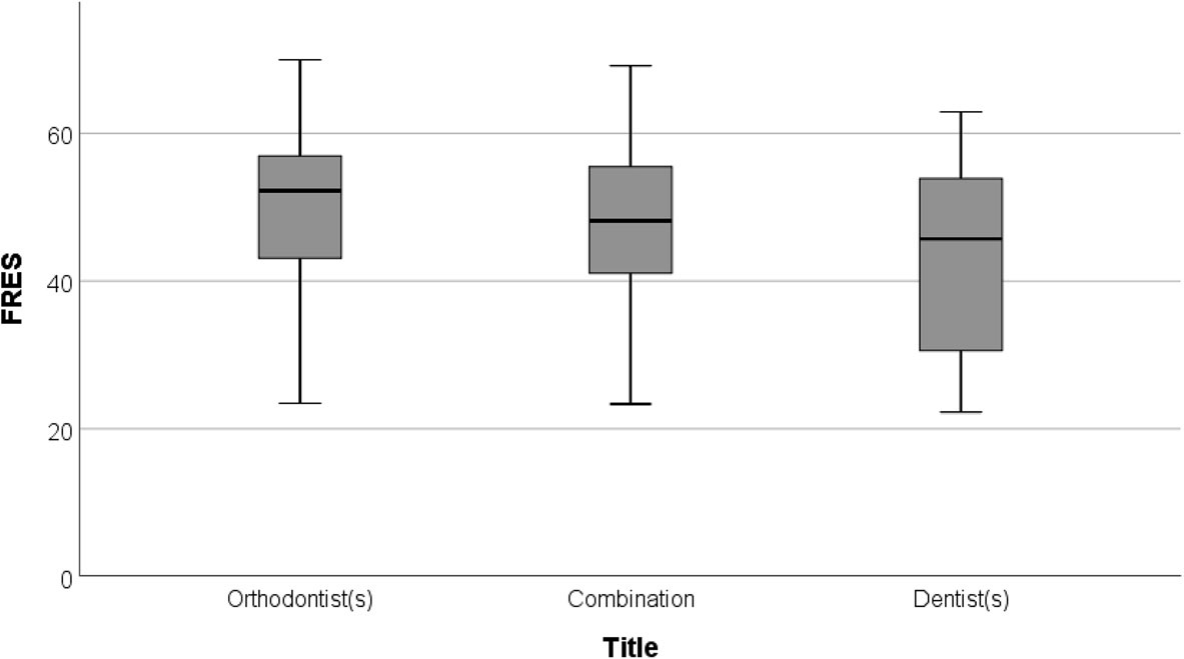
Box-and-whisker diagram illustrating the distribution of FRES of website information as classified by provider’s professional title

The mean FRES of the examined information text (48.2) was categorized as difficult to read or requiring college level. FRES of orthodontic websites was significantly higher than that of dental websites (*P* < 0.01), but both fell under the same reading difficulty category (Fig. 2).

Readability scores of websites of practices located in the 3 most densely populated provinces were significantly higher than those located in multiple provinces (*P* < 0.035). Multi-location practices were assigned a mean FRES of 35.4, which refers to difficult-to-read text or college-level (Table 3).

**Table 3:** FRES and BDC score means of website information and design, respectively (in parentheses, the standard deviations)

		Professional title			Practice location		
	Overall	Orthodontist	Dentist	Combination	NH, SH, U	Other provinces	Multiple provinces
FRES	48.2 (11.5)	50.6 (10.1)	43.0 (12.0)	46.8 (14.1)	49.1 (11.8)	48.9 (10.8)	35.4 (8.3)
BDC score	53.9 (12.3)	52.4 (11.3)	55.8 (13.7)	60.8 (13.0)	54.8 (12.8)	51.5 (11.0)	69.2 (9.7)

NH, SH, and U stand for the provinces of North Holland, South Holland, and Utrecht

#### Design

**Fig. 3:**
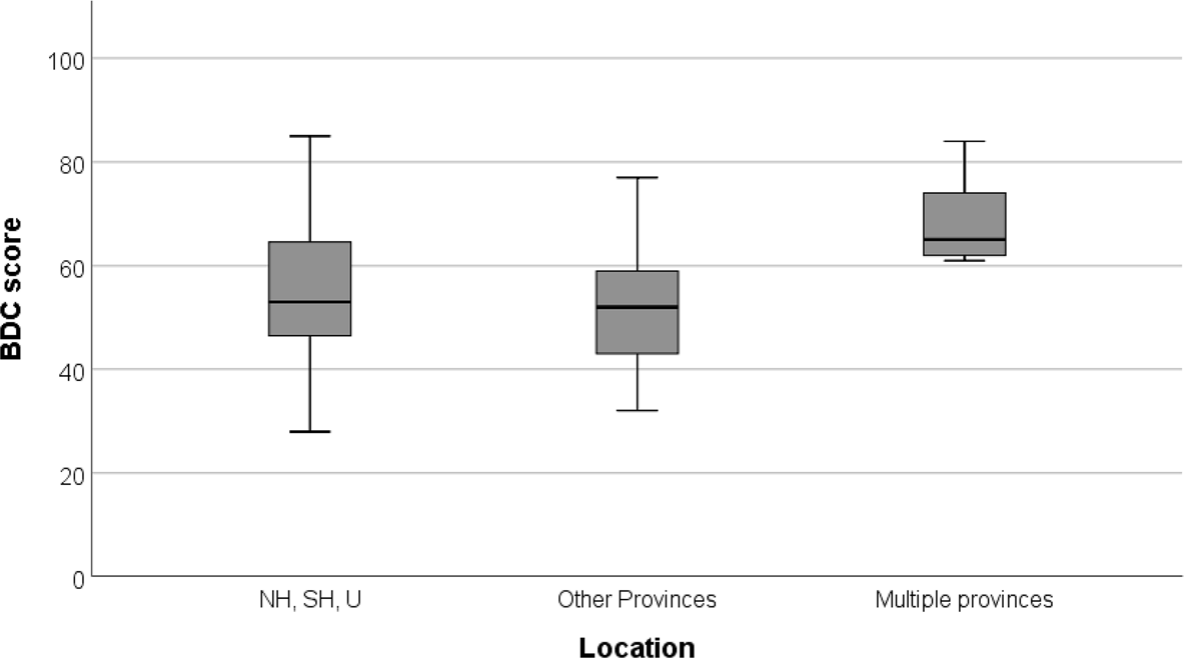
Box-and-whisker diagram illustrating the distribution of BDC scores of the websites as classified by practice location. NH, SH, U stand for the provinces of North Holland, South Holland and Utrecht

Two websites were not suitable for the application of the BDC tool and therefore excluded from the technical performance analysis. Websites of practices established in multiple provinces presented the highest mean BDC score (Table 3). BDC scores of these practices were significantly higher than those located in the 3 most densely populated provinces or other provinces (*P* < 0.006) (Fig. 3).

## Discussion

Ethical compliance of the websites of orthodontic and dental practices to the existing EU regulations has been so far indirectly investigated in the UK, using the GDC guidelines based on CED recommendations. These studies concluded that despite the progress made over time, UK orthodontic practice websites generally failed to comply with GDC instructions [2, 7–9]. In this sense, nearly 1 out of 10 orthodontic websites fully complied with the principles outlined by GDC [2].

According to the results of the present study, 26% of the examined Dutch websites were entirely compliant to CED mandatory items, with the provider’s registration number being specified though, in less than half of the cases. Professional registration details were reported at higher rates ranging from 53 to 84% of orthodontic practices examined elsewhere [2, 8, 9]. On the contrary, such information was suboptimally provided by the UK and Dutch dental practice websites, viz., 19 and 27%, respectively [6, 11]. With respect to CED discretionary information, a link to a professional association was most frequently lacking but the websites outpaced previous reports [2, 7–9].

Furthermore, the mean FRES of the orthodontic information available on practice websites was difficult to read and required college-level reading skills. Research suggests that patient education materials should be written at a maximum reading level of sixth grade, which corresponds to a FRES of 80, to be well understood by readers [20]. From this perspective, none of the websites achieved the recommended reading level.

The text on websites of orthodontists was significantly easier to comprehend compared to dentists and multi-disciplinary practices, but the average reading level was still higher than the recommended level. However, orthodontic populations are globally, predominantly consisted of children and adolescents who should be accordingly instructed on-site and online [21]. Likewise, health information on 80% of Dutch websites regarding a wide range of medical conditions has been found to be written on a reading level too difficult for individuals with low health literacy and deprived message elements that improve understanding of health materials [22]. Bearing in mind that 25% of the population in The Netherlands has inadequate health literacy, the potential implications of the readability results on processing online health information might be even more severe [23].

Hypothetically, high website quality might be expected by centrally located practices or the ones financially managed by multiple owners [14]. Websites of practices located in multiple provinces were assigned significantly higher BDC scores than the rest, indicating superior website optimization. Even though this type of practice was underrepresented in the study sample, it referred mostly to dental chains accounting for 148 practice locations across the country. Consolidation of dental care and the creation of groups of practices known as dental chains reflects a flourishing worldwide phenomenon in the healthcare industry [24]. In the USA, these large dental firms increased their establishments by 318% within a decade [25]. In Europe, consolidation is mostly evident in Finland, where dental chains hold 35% of the market in terms of the number of dentists. The ample resources of a dental chain and centralized back-office functions like shared IT service centers enable vertical integration of services such as advanced website design and support throughout the organization [24].

This study presents certain limitations that need to be acknowledged. Firstly, as in all cross-sectional observational studies, the sample of websites was collected at a single timepoint using a specific search strategy, and thus, interpretation of the findings should not be expanded. Nevertheless, the sample size can be considered relatively large and representative of the current orthodontic workforce in The Netherlands. Secondly, the content of the websites was evaluated in terms of adherence to ethical guidelines and readability, and not in terms of accuracy. As research on online orthodontic information has warned against the highly variable quality of materials related to treatment techniques, complications, and oral hygiene instructions, it would be useful to examine in the future the degree of agreement of the displayed text with the best available scientific evidence [26–30]. Thirdly, the evaluation of the technical design of the websites was performed by means of a rather new website assessment tool. Still, BDC is heavily used in business development strategies and provides an objective measure of the technical performance of a website.

The results of this multi-level website assessment advocate the need for the intensification of efforts to improve reporting of information in accordance with CED, simplify the context of the posted information, and optimize the technical design of the Dutch orthodontic practice websites. KNMT guidelines should be refined and monitored by domestic professional associations. Due to the dynamic nature of the Internet, regular review of the guidelines is necessary to keep pace with the constantly evolving technology [3]. In this direction, the close collaboration of EU with national health professional bodies may be helpful in facilitating transparent verification procedures like CE marking [31]. The informational content of the websites needs to be substantially revised to meet the reading standards of children and people with low health literacy. Inclusion of features that enhance information processing like animation, narration, and interactivity should be considered in developing appropriate orthodontic information materials [22]. Lastly, orthodontic practice websites may jointly cite easily understandable patient education materials approved by professional working groups and expert panels to ensure reading comprehension.

## Conclusions

- Despite the websites of Dutch orthodontic care providers comply to a large extent to the mandatory CED items, basic professional information like registration number and origin of education is commonly missing.
- The available orthodontic information requires advanced reading skills and should be therefore properly revised.
- A significantly more advanced website design was observed in multi-location practices.

## Data Availability

The datasets used and/or analyzed during the current study are available from the corresponding author on reasonable request.

## Abbreviations

CED: Code of Ethics for Dentists
EU: European Union
FRES: Flesch Reading Ease Score
GDC: General Dental Council
KNMT: Koninklijke Nederlandse Maatschappij tot bevordering der Tandheelkunde
NH: North Holland
NVvO: Nederlandse Vereniging van Orthodontisten
OVAP: Orthodontische Vereniging van Algemeen Practici
SH: South Holland
U: Utrecht
UK: United Kingdom
